# CREST – Classification Resources for Environmental Sequence Tags

**DOI:** 10.1371/journal.pone.0049334

**Published:** 2012-11-08

**Authors:** Anders Lanzén, Steffen L. Jørgensen, Daniel H. Huson, Markus Gorfer, Svenn Helge Grindhaug, Inge Jonassen, Lise Øvreås, Tim Urich

**Affiliations:** 1 Department of Biology and Centre for Geobiology, University of Bergen, Bergen, Norway; 2 Uni Computing, Uni Research AS, Bergen, Norway; 3 Centre for Bioinformatics, Tübingen University, Tübingen, Germany; 4 Fungal Genetics and Genomics Unit, AIT Gmbh and University of Natural Resources and Life Sciences, Tulln, Austria; 5 Department of Informatics, University of Bergen, Bergen, Norway; 6 Department of Genetics in Ecology, University of Vienna, Vienna, Austria; Natural History Museum of Denmark, University of Copenhagen, Denmark

## Abstract

Sequencing of taxonomic or phylogenetic markers is becoming a fast and efficient method for studying environmental microbial communities. This has resulted in a steadily growing collection of marker sequences, most notably of the small-subunit (SSU) ribosomal RNA gene, and an increased understanding of microbial phylogeny, diversity and community composition patterns. However, to utilize these large datasets together with new sequencing technologies, a reliable and flexible system for taxonomic classification is critical. We developed CREST (**C**lassification **R**esources for **E**nvironmental **S**equence **T**ags), a set of resources and tools for generating and utilizing custom taxonomies and reference datasets for classification of environmental sequences. CREST uses an alignment-based classification method with the lowest common ancestor algorithm. It also uses explicit rank similarity criteria to reduce false positives and identify novel taxa. We implemented this method in a web server, a command line tool and the graphical user interfaced program MEGAN. Further, we provide the SSU rRNA reference database and taxonomy SilvaMod, derived from the publicly available SILVA SSURef, for classification of sequences from bacteria, archaea and eukaryotes. Using cross-validation and environmental datasets, we compared the performance of CREST and SilvaMod to the RDP Classifier. We also utilized Greengenes as a reference database, both with CREST and the RDP Classifier. These analyses indicate that CREST performs better than alignment-free methods with higher recall rate (sensitivity) as well as precision, and with the ability to accurately identify most sequences from novel taxa. Classification using SilvaMod performed better than with Greengenes, particularly when applied to environmental sequences. CREST is freely available under a GNU General Public License (v3) from http://apps.cbu.uib.no/crest and http://lcaclassifier.googlecode.com.

## Introduction

Marker gene sequencing (also known as “barcoding” or “meta-barcoding) is an increasingly common technique for profiling the taxonomic composition and diversity of environmental samples. Facilitated by the rapid development of sequencing technologies, such studies are rapidly becoming routine and increasingly sophisticated. The technique has a clear potential for revolutionizing the field of microbial ecology as well as medical microbiology, by frequent and routine profiling of environmental as well as human microbiome samples. It has even been used in macro-ecology to monitor the distribution and dispersal of animal species [1]. For prokaryotes, the small-subunit ribosomal RNA (SSU rRNA) has become the *de facto* standard marker gene targeted by amplicon (or “tag”) sequencing [2]. However, the choice of marker, primers and marker region targeted varies among individual research laboratories and studies. In metagenomic or metatranscriptomic studies, sequences containing SSU rRNA or other markers can also be subjected to taxonomic profiling [3], [4], [5]. In either case, none of the existing “next-generation” sequencing protocols available allow for full-length sequencing of the SSU rRNA gene.

Pyrosequencing as developed by 454 Life Sciences (Roche) was the first high-throughput sequencing technology to be applied for sequencing of SSU rRNA. The current generation of pyrosequencing instruments (GS FLX+) can generate shotgun sequencing reads up to 800 bp long, while amplicon sequencing is only supported using the previous generation chemistry at the time of writing with read lengths of approximately 450 bp [6]. Other sequencing platforms gaining popularity are Illumina Hi-Seq, yielding read lengths of 100–150 bp [7], or 200 bp if assembly of paired-end reads is used as described by [8] or [9], and IonTorrent, yielding read lengths over 200 bp. Regardless of technology used, accurate taxonomic classification is of paramount importance to the interpretation of the resulting sequencing data [2]. The quality of results depends on read length, choice of taxonomic marker, region (the latter particularly important for shorter reads) [10], and last but not least on the classification method and taxonomy applied. Indeed, the quality of the taxonomy and reference database can have a more significant effect on results than the classification method [10].

In the last years, a large amount of SSU rRNA sequence data has been collected, organized and aligned in databases such as SILVA [11], the Ribosomal Database Project (RDP) [12] and Greengenes [13]. However, we are still only “scratching the surface” of global biodiversity with countless novel species and genera waiting to be discovered [14], [15], [16]. Many are also hidden among the hundreds of thousands of existing environmental sequences disguised under uninformative labels such as “uncultured bacteria” and thus remain without proper taxonomical descriptions. We can therefore expect that the Tree of Life representing our current understanding of the phylogeny of all living and extinct organisms, will receive many new branches and undergo many topological changes. Meanwhile, taxonomic classification will remain challenging. How to deal with the environmental sequences whose taxonomical affiliations remain unclear is a crucial consideration, as these unquestionably can be useful for classifying other similar environmental sequences. Including such environmental sequences in addition to cultured type strains may be crucial for phylogenetic or taxonomical work [17].

One challenging issue in taxonomical classification is polyphyletic taxa. Some of these, e.g. “Uncultured bacteria” are intentionally created as placeholders for sequences whose taxonomical affiliations are unclear. Others result from submission of sequences with incorrect taxonomical classification or incomplete knowledge of a phylogenetic group. Both categories can cause the classification sensitivity and resolution to drop [3]. Assignments to polyphyletic groups are also inherently less meaningful. Still, there are situations where taxa are well established in the literature, but known to be polyphyletic, e.g. *Clostridia* and *Bacilli* [18]. Removing them is not always desired until an alternative taxonomy has been established. Another challenging issue is identification of novel sequences. In order not to miss such potentially interesting information it is important to clearly be able to identify them, rather than assigning them incorrectly to an existing taxon [19].

To better deal with the above-mentioned issues, we present a set of resources for taxonomic classification that utilize environmental sequences together with reference strains. Branded as CREST (**C**lassification **R**esources for **E**nvironmental **S**equence **T**ags), we present a simple alignment- and lowest common ancestor (LCA) based taxonomic classification method, implemented as a web-server, command line tool and in a new version of the program MEGAN [20]. We also present a reference database and taxonomy for classification of environmental SSU rRNA sequences. This reference database, labeled *SilvaMod*, was derived using extensive manual curation from the taxonomically annotated SILVA Reference alignment (SSURef nr release 106) [11]. In addition, SilvaMod includes explicit rank information derived from the NCBI Taxonomy. A similar strategy was recently carried out by taxonomical annotation of Greengenes [21].

CREST is equally suitable for classification of sequencing data from SSU rRNA PCR amplicons as from shotgun metatranscriptome or metagenome sequences, not only from bacteria and archaea (prokaryotes), but also from eukaryotic taxa. We illustrate the performance of the databases and the assignment method, and compare this to the RDP Classifier [22] and SINA Aligner, both of which are commonly used methods for taxonomic classification of SSU rRNA sequences. We use two types of cross-validation; ten-fold, and removal of taxa, the latter to better simulate a situation where a novel taxon is discovered. We also apply the method to four environmental datasets generated using different sequencing technologies and compared the number of identified taxa and the proportion of classified reads at different taxonomical ranks. While originally developed for classification of SSU rRNA sequence data, CREST has wide applicability since it provides a framework for generating and utilizing custom taxonomies and reference databases. This procedure only requires a taxonomically annotated custom alignment, created e.g. with the program ARB [23].

## Results

### Overview of CREST

**Figure 1:**
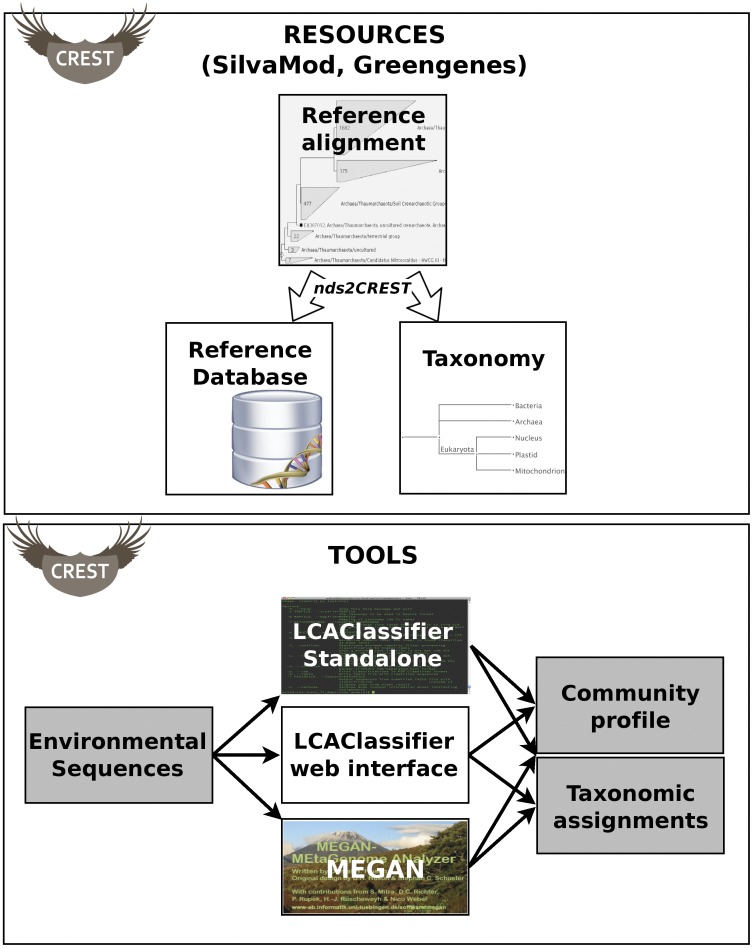
Overview of the resources of CREST. The flow of information during the construction of a new reference database (top part) or classification (bottom part) is represented by arrows. The classification tools MEGAN or LCAClassifier can utilize CREST taxonomy files and databases such as SilvaMod for classification of environmental sequences, aligned to the reference database with Megablast.

Figure 1 presents the resources of CREST, along with the flow of information during the construction of a new reference database (top part) or classification (bottom part). CREST includes:

1. the manually curated SSU rRNA taxonomy and reference database *SilvaMod* based on a modification of the taxonomical annotation used in SILVA SSURef nr release 106;
2. supplementary files for using the Greengenes taxonomy and database as an alternative;
3. a simple classification method based on pairwise alignment and assignment to the lowest common ancestor (LCA) of the resulting highest-scoring alignments;
4. implentations of the classification method as webserver and command line tool (LCAClassifier), and;
5. a new version of the program MEGAN [20] offering CREST classification.

The LCAClassifier and MEGAN implement the same classification algorithm and can use any taxonomy and CREST-compatible reference database and taxonomy, in addition to SilvaMod and Greengenes. Starting with a reference alignment of a taxonomic marker sequences from a collection of taxa, such databases can be derived with the script *nds2CREST* (see Methods and online technical documentation at http://code.google.com/p/lcaclassifier/wiki/Userguide for details).

### SilvaMod and Greengenes Reference Databases

Release 106 of the SILVA non-redundant SSURef database includes manual taxonomic annotations of its aligned sequence clusters, with a resolution up to genus rank. According to the developers, annotations were based on Bergey’s Manual of Systematic Bacteriology (vol. 1 to 4) [24], [25], [26], [27], the List of Prokaryotic names with Standing in Nomenclature [28], *Candidatus* taxa and names without standing in nomenclature (described in detail at http://www.arb-silva.de/documentation/faqs/). We curated these annotations and the taxonomical structure itself (the “SILVA Taxonomy”). in order to comply with recent phylogenetic work and to incorporate proposed environmental clades as suggested in a selection of phylogenetic studies (see Methods). Importantly, we only carried out such revisions if the resulting annotation agreed with the clustering in SSURef, in order to avoid inserting apparently polyphyletic taxa.

As the SILVA Taxonomy does not generally offer annotations for eukaryotic sequences at higher resolutions (order and above), we chose to instead use annotations to the NCBI Taxonomy for this domain. Annotations were manually verified and selected in order to avoid sequences whose taxonomic annotations were in conflict with the topology of the alignment-based tree (see Methods for details). To facilitate the identification and classification of plastid and mitochondrial 16S sequences as indicators of eukaryotic organisms, these were placed in the SilvaMod taxonomy as sub-domains together with the nuclear 18S rRNA directly under the eukaryotic domain (see box “Taxonomy” in Fig. 1), even though this strictly does not agree with the phylogenetic origin of these genes.

The resulting SilvaMod database contains 254,671 sequences from the SILVA SSURef nr release 106, along with curated taxonomical annotations including explicit rank information, formatted for MEGAN and the CREST LCAClassifier. Out of these, 72% are annotated to genus rank and 92% to at least family rank or better and the taxonomy contains in total 99 phyla and 39 candidate divisions. At higher resolution, there are 1,237 orders, 3,933 families and 9,906 genera including candidate divisions and environmental clades.

We also make available taxonomic annotations for the 2011 release of Greengenes [21], in the format accepted by MEGAN and the CREST LCAClassifier. This database contains more sequences (408,315) from a similar number of taxa at the phylum rank. However, the Greengenes taxonomy contains a much smaller number of taxa at higher resolutions, e.g. only 230 orders, 394 families and 1061 genera. One reason for this difference is the many eukaryotic taxa present only in SilvaMod.

### Classification Tools: MEGAN and the LCAClassifier

Alignment-based classification using LCA and minimum similarity filters (see Methods) was implemented in the CREST LCAClassifier and by extending MEGAN [20] (v4.68+). MEGAN uses a graphical user interface and can also export assignments, community composition and taxon-specific sequences in text format. In addition, the composition of two or more communities can be compared [29].

The CREST LCAClassifier uses a command line interface and reports community composition in a simple tab-separated text format allowing for overview of taxon abundance and richness (for amplicon sequences) at each taxonomic rank. Several alignment files, constructed using Megablast, can be classified simultaneously, which facilitates easy comparison between classification results from several datasets (by adding output for taxa present in at least one dataset to all). In addition, assignment information can be exported along with sequence data in FASTA format, or without it as comma-separated text. The CREST LCAClassifier is also available through a web interface at http://apps.cbu.uib.no/crest including the Megablast alignment step. The user simply uploads one or several sequence files in FASTA-format. A maximum of 1,000 sequences is currently enforced by the webserver, but exceptions may be granted on request.

Default values of LCA parameters were chosen conservatively based on cross-validation testing (see below). However, the appropriate parameters depend on the community studied and can be adjusted in the classification tools. See “Alignment-based classification – LCAClassifier” in Methods for a discussion of parameter choice.

In addition to LCA classification, we added a minimum similarity filter in order to decrease the false positive rate for “novel” or noisy sequences, with low similarity to reference sequences (see Methods). Using the CREST LCAClassifier, such sequences are flagged as “Unknown” members of the taxon to which they were assigned after filtering and can be retrieved from the FASTA- or assignment output data. Using MEGAN, or verbose output of the CREST LCAClassifier, information about all such assignments is written to the output dialog.

When analyzing data from amplicon libraries, an important first step is quality filtering followed by noise removal [30] or clustering [31], as well as chimera removal, in order to compensate for artifacts resulting from sequencing or PCR [32]. For pyrosequencing or IonTorrent sequence reads, we recommend using AmpliconNoise for pre-processing as this can remove more sequence noise than other available programs [30], [33] and ensures compatible annotation. Regardless of the method used, the result is a set of unique sequences, each representing a variable number of reads. For AmpliconNoise, the reads of each unique sequence are determined as likely to originate from identical nucleotide sequences. Alternatively, sequences representing similar reads in a cluster (OTU) can be submitted if the sequence names in the FASTA-file containing filtered sequences or OTU representatives are annotated with read abundance (using “*weight = N*” or “*_N*”), MEGAN or the CREST LCAClassifier will report both the weighted read abundance and number of unique sequences (i.e. richness) for each taxon. In addition, the CREST LCAClassifier calculates a Chao-estimate [34] of minimum richness for each taxon.

### Cross Validation Testing

To evaluate the performance of CREST with SSU rRNA sequences, we performed two types of cross-validation testing (a technique partitioning the reference dataset into subsets used for re-training and validation). Firstly, exhaustive ten-fold cross validation was used and secondly, removal of whole genera, families and phyla (see Methods for details). Tests were repeated with randomly cropped sub-sequences derived from these with lengths 450 and 100 bps; the approximate read lengths from the GS FLX Titanium (pyrosequencing) and Illumina Hi-Seq platforms, two of the most commonly used methods for high-throughput sequencing. Results from Megablast alignment followed by CREST LCAClassifier are hereafter referred to as “LCA”.

The same test regime was also carried out with the Greengenes database to compare effects of the two reference databases on classification results. To compare the performance with another popular method, the RDP Classifier [22], the same tests were carried out with this program using its default training dataset (v6/2.32). An alternative training dataset for the RDP Classifier using Greengenes was also evaluated, retraining the classifier via a QIIME script [35], designed intentionally to classify reads only to the family rank. This represents the recommended classification method for SSU rRNA QIIME.

**Figure 2:**
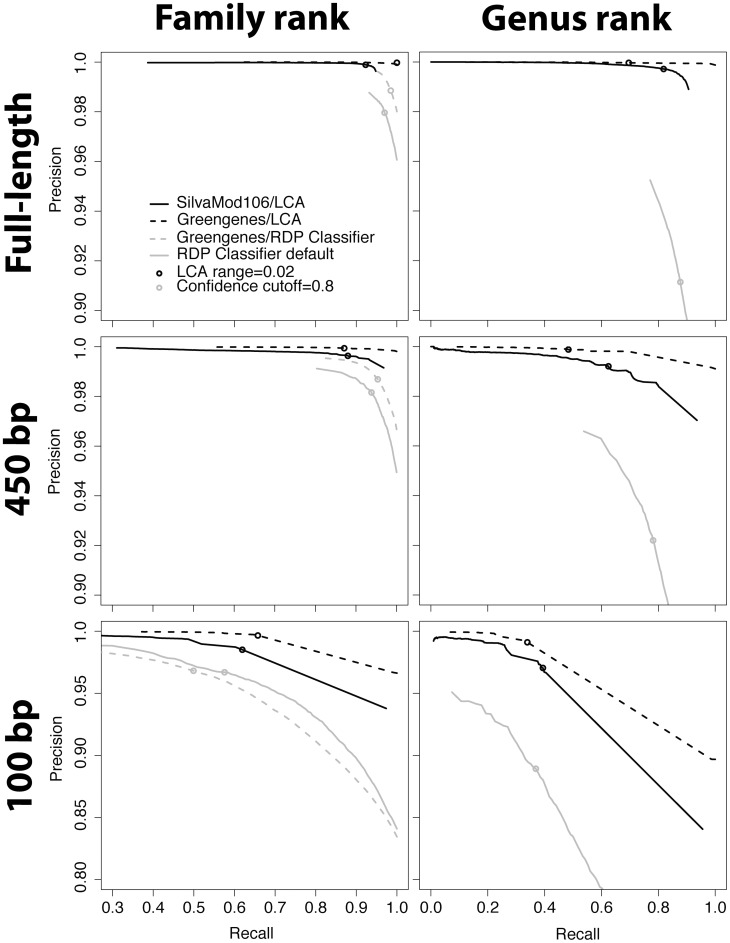
Precision-recall curves from ten-fold cross validation. Shows the precision (number of correct assignments/number of assignments made) on the y-axis and measured recall (sensitivity or true positive rate) on the x-axis, when varying LCA range or confidence cutoff. Circles indicate the default cutoffs (cutoff for RDP = 0.8, LCA range = 00.2).

Table 1 lists the resulting assignment accuracies (fraction of sequences classified correctly with default parameters) from ten-fold cross validation of the different classification strategies. SilvaMod with LCA performed best in five cases out of nine. Using the Greengenes database achieved slightly higher accuracy at family rank. Figure 2 shows the results from this test as precision-recall graphs, generated by varying the LCA range or confidence cut-off. This confirms that the CREST LCAClassifier was capable of classification with both a higher recall and precision, compared to the RDP Classifier. The RDP Classifier produced consistently higher false discovery rates (the fraction of all classifications made that were incorrect, or *1-precision*), up to 11% at the recommended bootstrap confidence cut-off at 0.8. For LCA it never reached above 3%. The minimum similarity filter contributes to reducing false assignments with about 30% at genus and family level for full-length sequences using the default LCA range.

**Table 1:** Assignment accuracy from ten-fold cross validation.

			Accuracy per rank^a^		
Method	Training/Reference set	Fragment length	Genus	Family	Phylum
LCA^b^	SilvaMod	F.L.^d^	**82%**	92%	**99.9%**
LCA^b^	SilvaMod	450 bp	62%	88%	**99.7%**
LCA^b^	SilvaMod	100 bp	**38%**	61%	**94%**
LCA^b^	Greengenes	F.L.^d^	69%	94%	99%
LCA^b^	Greengenes	450 bp	48%	87%	99%
LCA^b^	Greengenes	100 bp	33%	**65%**	**94%**
RDP^c^	Greengenes	F.L.^d^	–	**97%**	98%
RDP^c^	Greengenes	450 bp	–	**94%**	95%
RDP^c^	Greengenes	100 bp	–	49%	51%
RDP^c^	RDP v6	F.L.^d^	81%	95%	99%
RDP^c^	RDP v6	450 bp	**73%**	92%	98%
RDP^c^	RDP v6	100 bp	35%	56%	90%

a Assignment accuracy defined as number of correct assignments divided by the total number of sequences tested, given at three different ranks. The best values for each combination of rank and fragment length are indicated in bold.

b Classification using Megablast alignments and the CREST LCAClassifier within a 2% LCA range of the highest bitscore as well as percent similarity filters.

c Naïve Bayes classification using the RDP Classifier with a bootstrap of 0.8. With the Greengenes training set, RDP Classifier was run via the QIIME script assign_taxonomy, which does not classify sequences beyond the family level.

d Un-cropped full-length sequences from the reference or training dataset.

Results from the second test, removal of whole taxa, are presented in Table 2. In this test, the RDP Classifier performed better for shorter sequences (100 bp) and for removal of whole phyla, whereas the CREST LCAClassifier performed better with longer sequences for removal of families or genera.

**Table 2:** Assignment accuracy from removal-of-taxa cross validation.

Method	Training/Reference set	Fragment length	Accuracy^a^ at removed rank level for removal of:		
			Genera	Families	Phyla
LCA^b^	SilvaMod	F.L.^d^	**98%**	90%	7%
LCA^b^	SilvaMod	450 bp	77%	64%	27%
LCA^b^	SilvaMod	100 bp	81%	66%	76%
LCA^b^	Greengenes	F.L.^d^	85%	**99.8%**	37%
LCA^b^	Greengenes	450 bp	**90%**	**85%**	24%
LCA^b^	Greengenes	100 bp	87%	72%	71%
RDP^c^	Greengenes	F.L.^d^	–	57%	**85%**
RDP^c^	Greengenes	450 bp	–	83%	**92%**
RDP^c^	Greengenes	100 bp	–	**99%**	**99%**
RDP^c^	RDP v6	F.L.^d^	62%	62%	21%
RDP^c^	RDP v6	450 bp	75%	78%	89%
RDP^c^	RDP v6	100 bp	**93%**	92%	96%

a *Accuracy* defined as number of correct assignments divided by the total number of sequences tested, given at three different ranks. The best values for each combination of rank and fragment length are indicated in bold.

b Classification using Megablast alignments and the CREST LCAClassifier within a 2% LCA range of the highest bitscore as well as percent similarity filters.

c Naïve Bayes classification using the RDP Classifier with a bootstrap confidence cutoff of 0.8. With the Greengenes training set, RDP Classifier was run via the QIIME script assign_taxonomy, which does not classify sequences beyond the family level.

d Un-cropped full-length sequences from the reference or training dataset.

### Performance with Environmental Datasets

In order to evaluate the reference/training datasets and classification methods on “real life” environmental SSU rRNA datasets, the number of identified taxa and the proportion of classified reads were compared, using four different datasets (see Table 3). In addition to the approaches tested with cross validation, we also included the online SINA Aligner from SILVA in this test. Since submission to the online version of SINA was limited to a maximum of 500 sequences at the time of testing, the test could only be carried out with the one test dataset below this size. As opposed to the RDP Classifier, which utilizes nucleotide composition (“k-mers”), SINA is an alignment-based method like CREST. However, the sequences are compared directly to the reference alignment rather than using pairwise alignments.

**Table 3:** Datasets used for performance testing.

Dataset	Sequencing technology	Library type	Total SSU rRNA reads*
Lake Lanier	GS FLX Ti	Shotgun metagenome	558
Forest soil	GS FLX Ti	Shotgun metatranscriptome	51,202
Siberian soil	Illumina	16S rRNA amplicons	2,173
Hydrothermal mat	GS FLX Ti	16S rRNA amplicons	8,903

* Reads with a BLASTN alignment bitscore >50 to a sequence in SilvaMod.

**Figure 3:**
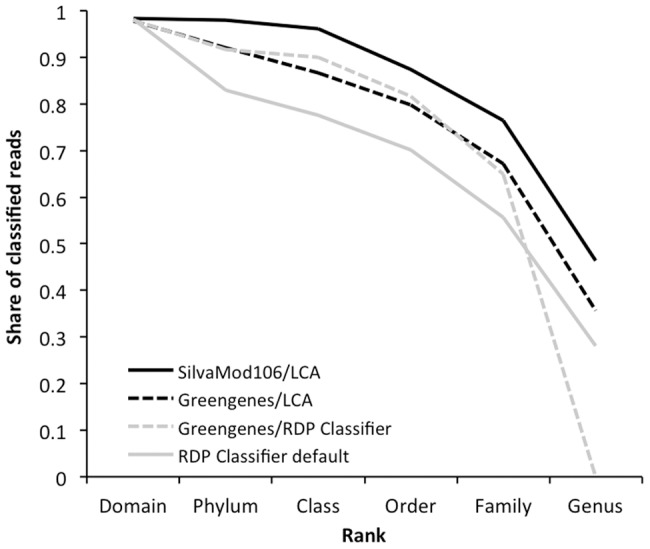
Average proportion of reads classified at different ranks in four environmental datasets. The CREST LCAClassifier (analogous to MEGAN) was tested using the full SilvaMod and Greengenes [21] reference databases with their respective taxonomies, as well as the RDP Classifier [22] retrained with Greengenes (99%OTU dataset; executed via QIIME) and version 6 of the default RDP training dataset.

SilvaMod with LCA performed best in terms of the share of reads classified for each environmental dataset and for each rank tested (phylum, family and genus), with only one exception (see Table 4). Compared to the RDP Classifier used with the default training, SilvaMod with LCA managed to assign on average about 50% more reads at family level. With this classification approach more taxa were also detected in each dataset and rank, in all cases but two (Table 4). Figure 3 shows the average share of classified reads in the four datasets, with SilvaMod and LCA showing the highest values across all ranks, followed with Greengenes, giving similar sensitivity with LCA and RDP Classifier assignment (except at genus level).

**Table 4:** Results from performance testing using environmental datasets.

			Share of reads assigned^a^			Unique taxa (B+A+E)^b^		
Method	Training/Reference set	Dataset	Genus	Family	Phylum	Genera	Families	Phyla
LCA^c^	SilvaMod	Lake Lanier	**36.2%**	**73.7%**	**99.5%**	**31+0+1**	**45+0+2**	**11+0+2**
LCA^c^	SilvaMod	Forest soil	**30.4%**	**69.8%**	**99.1%**	**232+0+166**	**156**+**1**+**167**	**29+2+46**
LCA^c^	SilvaMod	Siberian soil	31.2%	**69.5%**	**93.6%**	**51+1+0**	**81+1+0**	**20+1+0**
LCA^c^	SilvaMod	Hydrothermal mat	**87.5%**	**93.0%**	**99.6%**	**36+2+1**	**42+8+1**	19+2+1
LCA^c^	Greengenes	Lake Lanier	11.5%	64.5%	98.9%	15+0+0	25+0+0	**13+0+0**
LCA^c^	Greengenes	Forest soil	14.7%	55.1%	84.1%	130+0+0	126+1+0	31+2+2
LCA^c^	Greengenes	Siberian soil	**39.0%**	60.1%	85.6%	38+1+0	53+1+0	18+1+0
LCA^c^	Greengenes	Hydrothermal mat	77.5%	89.0%	99.4%	15+1+0	23+6+0	**21+2+1**
RDP^d^	Greengenes	Lake Lanier	0	72.2%	91.8%	0	28+0+0	9+0+0
RDP^d^	Greengenes	Forest soil	0	52.2%	86.7%	0	111+0+0	16+2+1
RDP^d^	Greengenes	Siberian soil	0	53.4%	90.5%	0	53+1+0	10+1+0
RDP^d^	Greengenes	Hydrothermal mat	0	81.6%	97.8%	0	19+3+0	9+2+0
RDP^d^	RDP v6	Lake Lanier	9.3%	51.1%	87.1%	17+0+0	20+0+2	10+0+2
RDP^d^	RDP v6	Forest soil	11.9%	40.4%	80.9%	176+2+0	95+2+0	20+2+1
RDP^d^	RDP v6	Siberian soil	6.7%	39.7%	66.0%	36+1+0	39+1+0	10+1+0
RDP^d^	RDP v6	Hydrothermal mat	84.4%	91.7%	97.7%	21+2+0	17+2+0	8+2+0
SINA^e^	SSURef108	Hydrothermal mat	20.4%	27.7%	93.2%	32+1+0	25+5+0	9+2+0

a Proportion of the total reads in the dataset for which taxonomical assignment was achieved at the given taxonomical level.

b Number of unique taxa identified given separately for bacteria + archaea + eukaryotes. Where the highest total number of taxa was predicted from a test dataset, the number is indicated in bold.

c Classification using Megablast alignments and the CREST LCAClassifier within a 2% LCA range of the highest bitscore as well as percent similarity filters.

d Naïve Bayes classification using the RDP Classifier with a bootstrap confidence cutoff of 0.8. With the Greengenes training set, RDP Classifier was run via the QIIME script assign_taxonomy.

e LCA clasification based on SINA Aligner, using default parameters at SILVA website.

Classifications of the most abundant taxa agreed well, with a few exceptions. Sequences that were classified as *Sporichthyaceae* order *Frankinales* (21% abundance in the Lake Lanier dataset) using SilvaMod, were instead classified as *Actinomycetales* clade “ACK-M1” using Greengenes. Similarly *Oryzihumus* (22% in the Siberian soil dataset, according to SilvaMod) was classified as *Phycicoccus* according to Greengenes.

### Resource Requirements and Execution Time

Running Megablast and LCA classification of 1,000 SSU rRNA sequences from the Forest Soil dataset took less than 12 minutes using a quad-core Apple MacBook Pro with a 2 GHz Intel i7. As a comparison, the RDP Classifier trained with Greengenes used 2 minutes on the same dataset and MacBook while the SINA Online Aligner used 8 minutes. Given that 1,000 sequences is a moderately high richness after AmpliconNoise analysis, this allows for classification in a very reasonable time of amplicon libraries sequenced with pyrosequencing or IonTorrent and subjected to AmpliconNoise prior to classification. Since typically only about 0.1% of shotgun metagenome sequences contain the SSU rRNA gene [4], this is also a very reasonable time for classification of such datasets. However, it requires a pre-screening to select the SSU rRNA containing sequences before classification, for which there are several alignment and compositional based tools available faster than Megablast, such as Metaxa [36] HoSeqI [37] and USEARCH [38].

## Discussion

Ten-fold cross validation tests indicate that CREST LCA Classification achieves better recall with higher precision compared to the RDP Classifier and SINA, regardless of reference database (SilvaMod or Greengenes). In general, CREST also gives a higher recall and precision when using the Greengenes database compared to SilvaMod at family rank. This could indicate that the Greengenes taxonomy is more robust, i.e. contain fewer polyphyletic or incorrectly affiliated taxa. On the other hand, the Greengenes reference database contains more sequences and fewer taxa. Ten-fold cross-validation is expected to underestimate the prediction accuracy for both small and more complex reference datasets, as the taxonomic redundancy will be lower, or in other words each taxon is represented by fewer sequences. Considering this bias, results indicate that both databases perform comparatively well using CREST LCA, whereas the RDP Classifier gives rise to more false positives, especially at genus rank.

Removal of whole taxa is less biased to differences in reference database size and showed contrasting results compared to ten-fold cross validation. As a consequence of lower sensitivity, the RDP Classifier always performed better when classifying shorter sequences. It also performed better for phylum removal, when using Greengenes as training dataset. This corresponds to a situation where a previously un-encountered clade at “phylum level” is discovered (SSU rRNA sequence similarity typically <85%). The alignment and LCA based method struggled with this, as partial alignments were often produced and thus an assignment to the closest related phylum was made. A majority of sequences with more realistic novelty are correctly identified as unknown by the minimum similarity filter of MEGAN or the CREST LCAClassifier. In addition, these can be extracted from the dataset for further phylogenetic study. We recommend caution when interpreting the existence of such taxa, however, particularly for amplicon sequences with low abundance (such as “singletons”) or for shotgun sequencing reads, as these can represent sequencing or PCR artifacts rather than true biological novelty.

Using actual environmental datasets, the CREST LCAClassifier with SilvaMod consistently demonstrated an ability to provide more detailed taxonomic classifications than the other approaches tested, in terms of both number of reads assigned and number of taxa detected. These results indicate a stronger sensitivity at all three taxonomic levels tested (phylum, family and genus), both quantitatively (number of reads classified) and qualitatively (number of taxa recognized). As demonstrated using cross-validation, incorrect assignments are not likely to have influenced this test. The fact that eukaryotic 18S rRNA sequences were only present in SilvaMod contributed to these results but cannot explain them alone; more bacterial and archaeal taxa were also consistently predicted. In general, SSU rRNA offers limited resolution as a taxonomic marker for eukaryotic sequences, particularly at species level, why internal transcribed spacers (ITS) or large subunit (LSU) rRNA is often utilized instead [39], [40]. Similarly to our results using SSU rRNA, it has been shown that LCA classification as implemented in MEGAN yields better accuracy also for ITS [40] or LSU [41] rRNA sequences. CREST allows for the creation of such reference databases for the CREST LCAClassifier, from an alignment of ITS or LSU sequences.

The SINA Aligner performed significantly worse than all other methods on the one dataset it was tested on. However, the results of the comparison should be interpreted with some care; it may be that the default parameters used are more conservative than those used for the other methods. This is hard to estimate for SINA since it was not practically possible to include the method in the cross validation. It would have required the complete reference alignment to be re-built for each test for a fair comparison.

At the family rank, recall rates from the ten-fold cross-validation (Table 1) are roughly similar at family level to the fraction of sequences classified from the environmental amplicon datasets (Hydrothermal mat and Siberian soil) of corresponding lengths (450 bp and 100 bp, respectively). This indicates that the ten-fold cross-validation allows quite realistic testing of the methods at this resolution. At genus level, however, recall rates were consistently higher in the ten-fold cross-validation, probably because a large fraction of the reads in the environmental datasets belong to taxa that remain to be taxonomically described at this resolution.

In conclusion, CREST provides for efficient and accurate taxonomic classification of environmental sequence tags, i.e. those containing a suitable taxonomic marker, such as SSU rRNA. We propose a classification scheme with Megablast used for alignment to the proposed SilvaMod reference database, and an extended algorithm for Lowest Common Ancestor classification, as implemented in MEGAN and the CREST LCAClassifier. This results in higher classification rates than with existing taxonomically annotated reference databases such as Greengenes. As shown using environmental datasets as well as cross-validation, it also outperforms the RDP Classifier, regardless of training dataset used, both in terms of recall and false positive rate.

In addition to classification with the SilvaMod reference database, CREST-compatible configuration files for the Greengenes database are available at the CREST website (http://apps.cbu.uib.no/crest). By using both databases for prokaryotic sequences, a best practice is ensured and differences can be identified and manually studied in more detail. Using the *nds2CREST* script distributed with the CREST LCAClassifier, new CREST reference databases can also be made from a taxonomically annotated alignment-based tree in ARB-format, from any taxonomic marker (e.g. *rpoB*, LSU rRNA or ITS).

## Methods

### Constructing the SilvaMod Taxonomy and Reference Database

The non-redundant SILVA SSURef release 106 was downloaded in ARB-format from the SILVA website at http://www.arb-silva.de. Using the ARB software package [23], we removed all sequences with a pintail score below 75, alignment quality score below 75 or length below 1,200 bp, in order to retain only high quality sequences. Further, we revised the taxonomy of several bacterial and archaeal taxa. The most significant improvements update the taxonomy of the Archaea to include the proposed phylum *Thaumarchaeota* [42], [43], the *Actinobacteria* to comply with Bergey’s Taxonomic Outline [44], the *Acidobacteria* to incorporate proposed subgroups [45] and the *Cyanobacteria* to comply with the CyanoDB [46] and in some cases specific studies (details given in Supplementary Table S1). Other added taxa include the *Zetaproteobacteria* [47], *Rubritaleaceae* [27] and *Armatimonadetes* [48]. In addition, we identified a number of taxa whose taxonomic annotation disagreed strongly with the topology of the SSURef alignment-based tree and appeared poorly supported by phylogenetic studies. These were either re-assigned to existing parent taxa or novel ones labeled *incertae sedis*. Unique taxon names were always used and to this end we added the name of the only child taxon to several unlabeled or undetermined taxa, or removed them.

Annotations of the eukaryotic taxa using the NCBI Taxonomy were taken from the SSURef database and manually verified in order to remove all sequences where taxonomical affiliation was in clear conflict with the topology of the alignment-based tree. Selection of fungal reference sequences was done according to recent phylogenetic work [49], [50].

All manual changes are listed in Supplementary Table S1, which can also be downloaded as a text file from http://services.cbu.uib.no/supplementary/crest/and is using an unambiguous format that can be parsed by the *nds2CREST* script (see below). In total, 82 new taxa were added, 123 were renamed and 17 deleted. All sequences remaining after curation were exported in FASTA format. During this procedure, sequences were cropped so as only the part corresponding to the SSU rRNA gene was saved. This was achieved by applying the *Escherichia coli* positional filter in ARB, selecting alignment column 1,o00 and 43,183. A tab-separated text file listing the accession numbers and taxonomic placements of each sequence was exported (using “NDS export”).

We developed the python script *nds2CREST* distributed together with the CREST LCAClassifier in order to convert the exported sequence and taxonomic data from ARB into configuration files for MEGAN [20] and the CREST LCAClassifier. This script also reads a text version of the Manual Changes File (MCF; Supplementary Table S1). For each change specified in the MCF, it confirms that the change was properly carried out. In addition, the script removes all sequences without valid taxonomical annotation or specified to be removed in the MCF. After this procedure, it assigns taxonomic ranks for each taxon based primarily on the NCBI Taxonomy, where such information is available; secondarily on the name of the taxon using the suffices “-*ales*” and “-*acaea*” to indicate family or order level, respectively; and lastly based on the parent rank. The output of *nds2CREST* is (1) a tree-file in Newick format describing the topology of the taxonomy, (2) a tab-separated “mapping file” specifying the name and rank for each taxon, and (3) a reference sequence database in FASTA-format. In addition to SilvaMod, we also prepared such files from the Greengenes Taxonomy [21] using the same procedure, however without manual curation or positional filtering.

### Alignment-based Classification - LCAClassifier

Taxonomical classification of environmental sequences starts with alignment to a reference sequence database (such as SilvaMod or Greengenes) using the NCBI *blastall* implementation of Megablast, with default settings except (optionally) restricting the output to 100 alignments to save disk space and calculation time, and deactivating the low complexity filter (the latter was not used during testing but has negligible impact on SSU rRNA alignments). BLASTN was also evaluated as an alternative, but as we did not notice an increased performance relative to the faster Megablast, we do not recommend it for SSU rRNA classification. The CREST LCAClassifier requires that Megablast output is saved in XML format, whereas MEGAN [20] can also parse the plain text output.

The classification is then carried out based on a subset of the best matching alignments using the Lowest Common Ancestor (LCA) of this subset, as previously described in MEGAN [20]. Briefly, the subset includes sequences that score within x% of the “bit-score” of the best alignment, providing the best score is above a minimum value. We selected a minimum bit-score of 155 and an LCA range (x) of 2% as default parameters based on results from ten-fold cross-validation testing of SilvaMod, which resulted in relatively few false positives regardless of fragment length at the cost of slightly decreased recall. Lowering the LCA range increases the sensitivity at the cost of reduced precision, equivalent to moving to the right along the precision-recall curves of Figure 2, which can provide some guidance for selecting appropriate LCA range with different sequence lengths (note however, that the cross-validation testing only provides a rough indication of true precision and recall values). The appropriate LCA range also depends on the community studied. For example, the LCA range can be decreased to 1% if most sequences are similar to well-known type strains, or with longer read lengths (e.g. from Sanger sequencing or GS FLX+). Minimum bit-score has less effect on performance but we recommend increasing it when classifying amplicon sequences with longer read lengths, to e.g. 300 for FLX+.

The minimum similarity filter is based on a set of rank-specific requirements. Firstly, a sequence must be aligned with at least 99% nucleotide similarity to the best reference sequence in order to be classified to the species rank. For the genus, family, order, class and phylum ranks the respective cut-offs are 97%, 95%, 90%, 85% and 80%. These values were based on minimum similarities between closest neighbor SSU rRNA sequences inside the same taxa [51] then modified to further increase classification accuracy based on initial cross-validation testing. The filter ensures that classification is made to the taxon of the lowest allowed rank, effectively re-assigning sequences to parent taxa until allowed. Sequences with best-scoring alignments below the minimum bit-score are treated as unclassified and not analyzed by this filter.

The CREST LCAClassifier was implemented in Python (http://www.python.org) and can be executed on all major platforms.

### Performance Evaluation Using Cross-validation and Environmental Data

We performed exhaustive ten-fold cross-validation by randomly splitting the SilvaMod database into ten different sequence subsets of equal size. Each subset was then aligned to a concatenation of the other nine using Megablast and classified using the CREST LCAClassifier, in addition determining the LCA range at which the sequence could no longer be classified to each rank level. The default minimum bit-score (155) was also applied. Each test dataset was also randomly cropped into sub-sequences of 100 bp and 450 bp and the two resulting cropped subsets aligned. The same procedure was carried out for the Greengenes database and RDP Classifier default training dataset version 6 [22]. Instead of alignment, a re-training of the RDP classifier (v 2.3) was performed. Ten-fold cross-validation was also carried out on the Greengenes based training set *gg_99_otus_4feb2011* using RDP Classifier v.2.2 through the QIIME (v.1.4.0; [35]) script *assign_taxonomy.py*.

We also performed cross validation based on removal of whole genera, families and phyla. Testing was carried out using the four described reference (or training) datasets and was exhaustive, except for genera, where 100 genera were chosen randomly. The sequences of each taxon were aligned to a reference dataset with the sequences from that taxon missing. For the RDP Classifier tests, it was retrained in an analogous manner.

Assignments from cross validation tests were summarized using a custom python script and R (http://r-project.org). For each test, classification strategy, rank, and confidence cut-off (or LCA range), we calculated the number of true positives (*TP*; sequences correctly classified to a taxon), true negatives (*TN;* correctly unclassified), false positives (*FP;* incorrectly classified) and false negatives (*FN;* incorrectly unclassified). Assignment accuracy, precision and recall (sensitivity) was calculated using:
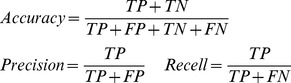



In addition to cross-validation tests, the following four environmental datasets (summarized in Table 3) were used:

1. SSU rRNA gene-containing pyrosequencing reads from the metagenome of Lake Lanier [5],
2. SSU rRNA-containing pyrosequencing reads from the metatranscriptome of an Austrian forest soil (unpublished),
3. Illumina-sequenced amplicon reads of the V4 region of 16S rRNA from Siberian tundra soil (unpublished), and
4. De-noised unique amplicon sequences of the V5–V6 region of 16S from a deep-sea hydrothermally associated microbial mat [4]

In addition to the mentioned classification strategies, dataset #4 was submitted to LCA-based classification with the SINA Aligner Online at the SILVA website (www.arb-silva.de/aligner) using default parameters and SSURef release 108 as reference alignment. Initially the Hidden Markov Model-based program SSuMMo [52] was also included in the comparison, using its SILVA v108 reference dataset and default parameters. However, limitations of the program’s output format made it impossible to calculate the number of assigned reads from classification of amplicon sequences representing several reads. Initial results for Environmental dataset 1 showed a consistently lower assignment rate at family level (35%) compared to other methods tested (51–74%) and a calculation time over ten times that of CREST (89 minutes for 1,000 sequences).

### Data Access

All resources including databases, taxonomy files and the source code for the CREST LCAClassifier and its web server are available under a GNU General Public Licence (v3) from http://apps.cbu.uib.no/crest and http://lcaclassifier.googlecode.com. Technical documentation describing how to install and use the program is available on the same websites. MEGAN can be downloaded from http://ab.inf.uni-tuebingen.de/software/megan/along with detailed documentation. All datasets used for testing can be downloaded from http://services.cbu.uib.no/supplementary/crest/.

## Supporting Information

Table S1 **Manual changes done during curation of the Silva Taxonomy (release 106) to SilvaMod.**
(XLSX)
